# Risk stratification of gastric cancer screening in community population based on oral contrast-enhanced ultrasonography examination: A 3-year follow-up analysis report

**DOI:** 10.3389/fonc.2023.1218800

**Published:** 2023-10-30

**Authors:** Sainan Guan, Ronghua Yan, Xiaomin Chen, Weiqiang Chen, Xi Zhou, Minghui Zhou, Zhengneng Xie, Wen Tan, Yongyan He, Juan Fu, Fan Yuan, Erjiao Xu

**Affiliations:** ^1^ Department of Medical Ultrasonics, The Eighth Affiliated Hospital of Sun Yat-sen University, Shenzhen, Guangdong, China; ^2^ Department of Radiology, The Eighth Affiliated Hospital of Sun Yat-sen University, Shenzhen, Guangdong, China; ^3^ Department of Radiology, Peking University Shenzhen Hospital, Shenzhen, Guangdong, China

**Keywords:** gastric cancer, oral contrast-enhanced ultrasonography, risk stratification, screening, thickness of the gastric wall

## Abstract

**Objective:**

This study aimed to retrospectively investigate the use of oral contrast-enhanced ultrasonography (O-CEUS) in assessing the thickness of the gastric wall for gastric cancer (GC) screening and to establish screening strategies for GC with different risk stratifications based on the gastric wall thickness.

**Methods:**

From January 2015 to March 2020, people who underwent O-CEUS at the Physical Examination Center of our hospital with at least three years of follow-up were included in this study. The thickness of the gastric wall measured by O-CEUS was divided into three groups using 6 mm and 9 mm as cutoff values. The occurrence of GC in each group was observed. The imaging and clinical information of these populations were recorded and analyzed. Kaplan–Meier survival analysis and Cox’s proportional hazards regression were performed to calculate the risk of GC occurrence.

**Results:**

A total of 4,047 people were finally included in this study. During the follow-up period, GC occurred in 7 individuals (incidence rate 0.17%). Among them, according to the thickness of the gastric wall, one case occurred in Group A (< 6 mm), two cases occurred in Group B (6-9 mm), and four cases occurred in Group C (>9mm). Based on Kaplan–Meier survival analysis, the curves of the three groups were significantly different (P < 0.01). The risk of GC occurrence in Group C and Group B were higher than that in Group A (4.76E+2-fold and 1.50E+2-fold).

**Conclusion:**

O-CEUS is a convenient, economical, safe, and noninvasive screening method for GC. Measuring the thickness of the gastric wall is helpful to predict the risk of GC occurrence according to our stratification screening system.

## 1 Introduction

According to the latest report, gastric cancer (GC) has the fifth-highest incidence rate and the third-highest mortality rate worldwide among all malignant tumors (1). Moreover, nearly half of the global incident cases of GC and deaths as a result of GC occur in China. Unfortunately, more than 90% of these incident cases are advanced GC (2). Therefore, early screening for GC is particularly important (3).

Generally, gastroscopy is the major screening modality for GC and has become a regular recommendation for people older than 40 years in Japan and South Korea (4, 5). However, gastroscopy for GC screening has not been popularized in China due to its invasiveness. Meanwhile, the popularization of gastroscopy is also limited by insufficient equipment, high cost for the examinations and the lack of skilled endoscopists.

Oral contrast-enhanced ultrasonography (O-CEUS) has become increasingly popular for the evaluation of gastric disease. O-CEUS is performed with the oral administration of a hyperechoic contrast agent with good sound permeability to fill the gastric cavity. The gas and contents in the gastric cavity were eliminated to improve the observation of the gastric cavity and gastric wall. As a noninvasive, painless, low-cost, convenient and real-time imaging technique, O-CEUS is more easily accepted by people and is suitable as an imaging modality for primary GC screening. Previous studies have suggested that O-CEUS is helpful for the detection of submucosal lesions of the stomach, the assessment of the degree of GC infiltration, and the preoperative staging of the advanced GC (6–10). Some recent studies have shown that the thickness of the gastric wall might be a valuable index for GC screening (11–15). However, as far as we know, there are fewer studies with long-term follow-up and large samples to evaluate the value of O-CEUS for GC screening.

Thus, in this study, we aimed to retrospectively analyze the use of O-CEUS to assess the thickness of the gastric wall for GC screening in a population undergoing physical examination and establish screening strategies for GC with different risk stratifications based on the thickness of the gastric wall.

## 2 Methods

### 2.1 Study subjects

From January 2015 to March 2020, people from companies and institutions in Shenzhen who were assigned to undergo physical examination at the Physical Examination Center of the Eighth Affiliated Hospital of Sun Yat-Sen University and underwent O-CEUS examination were included in this study. All populations underwent physical examinations, including O-CEUS, at our hospital. And then they received regular physical examinations, including O-CEUS or gastroscopy, at our hospital or other hospitals for at least three years. Individuals with a history of previous gastric cancer or gastric surgery, or people without complete clinical and imaging information, or people lost to follow-up were excluded.

This research has been approved by the Institutional Review Board of our hospital (No. 2022-055-01). As a retrospective study, the informed consents of all participants were waived in this study.

### 2.2 Instrument and contrast agent

The oral ultrasound contrast agent (O-UCAs) used for O-CEUS in this study was Tian-Xia^®^ (Dongya Pharmaceuticals Co., Huzhou, China). The O-UCAs (about 50 grams) were dissolved in 500 ml of warm water for O-CEUS preparation. It is a cellulose-based agent with a pleasant taste (6).

Eight different ultrasound machines were used for O-CEUS. They were Logiq E9 (GE Healthcare, Chicago, USA); Voluson E10 (GE Healthcare, Chicago, USA); iU elite (Philips, Amsterdam, Netherlands); Mylab 90 (Esaote, Genoa, Italy); DC-60, DC-8, DC-80 (Mindray, Shenzhen, China) and Arrieta 60 (Hitachi, Tokyo, Japan). The respective convex array probes were used with frequencies 3-5 MHz.

### 2.3 O-CEUS Procedure

All people fasted for 8 hours before O-CEUS to ensure that the gastric cavity was empty. Before O-CEUS examination, the people were asked to drink all 500 ml of the O-UCAs solution at once.

The scanning procedures were standardized and unified in our center and could be divided into three specific steps.

**Figure 1:**
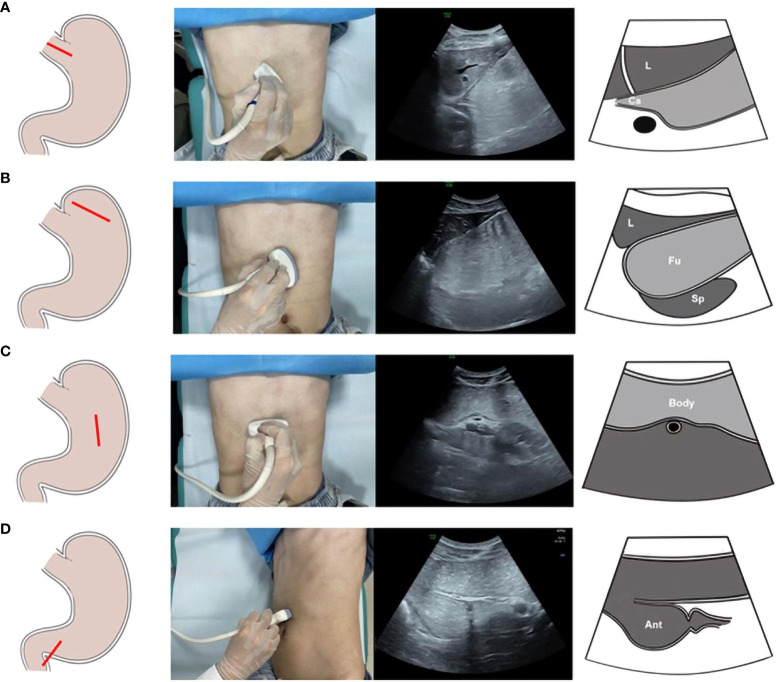
The scanning procedures of Oral contrast-enhanced ultrasonography. Ultrasonic scanning plane of the gastric cardia **(A)**. Ultrasonic scanning plane of the gastric fundus **(B)**. Ultrasonic scanning plane of the gastric body **(C)**. Ultrasonic scanning plane of the gastric antrum **(D)**.

Step 1: The people lay on his or her back, and the probe was placed below the xiphoid process and then moved to the left costal arch to scan the cardia. Afterward, the probe was moved to the left intercostal space to observe the gastric fundus. Subsequently, the people lay on his or her right side to allow the O-UCAs to flow along the gastric cavity. Cross-sectional scanning was performed to observe the gastric fundus, gastric body and gastric antrum from left to right. Finally, the person lay on his or her back, and the gastric antrum and pylorus were scanned (**Figure 1**).

Step 2: The people lay in the right lateral position, and the probe scanned along the transverse axis of the gastric cavity from right to left.

Step 3: The people remained lying in the right lateral position, and the probe scanned along the longitudinal axis from the proximal to the distal gastric cavity.

Steps 2 and step 3 were mainly performed to repeat scanning to reduce missed diagnoses.

### 2.4 Collection of images data and clinical information

During the O-CEUS examination, the filling state of various parts of the gastric cavity was observed. For people who had found focal gastric wall thickening (where the tumor or abnormality was located), the thickness of the gastric wall at the thickest point was chosen to measure. For people who had not found focal gastric wall thickening, according to the previous studies (13, 15, 16), the thickness of the gastric wall of the antrum (where gastric ulcers and GC often occurred) was measured on the long-axis section of the gastric angle when the antrum was fully filled and the gastric wall was contracted.

Meanwhile, the imaging information of O-CEUS and clinical information of enrolled people were recorded, including age, gender, calcification on the gastric wall, and the continuity and integrity of the 5-layer structure of the gastric wall.

### 2.5 Statistical analysis

Kaplan–Meier survival analysis was used to analyze the endpoint event (GC occurrence). Cox proportional hazards regression analysis was performed to examine the correlation between the gastric wall thickness grouping and gastric cancer events. The “survival analysis” software package SPSS v27.0.0 was utilized to calculate log-rank P values, hazard ratios (HRs), and 95% confidence intervals (CIs). In addition, the survival differences were visualized by generating Kaplan–Meier survival plots.

According to the literature and our previous research experience, the gastric wall thickness measured by O-CEUS was divided into three groups: 1) Group A: < 6 mm, 2) Group B: ≥6 mm but ≤ 9 mm, and 3) Group C: > 9 mm. The occurrence of GC was observed in each group (17).

## 3 Results

### 3.1 Basic information about the enrolled population

**Figure 2:**
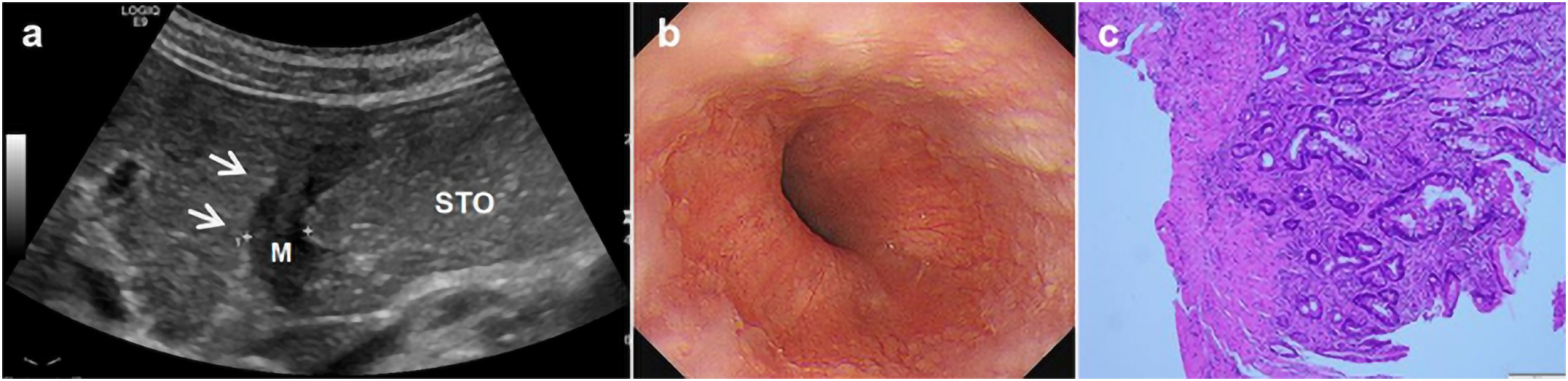
A 69-year-old man underwent physical examination in our hospital. Oral contrast-enhanced ultrasonography showed a gastric wall thickness of 12.1 mm in the gastric body and diagnosed as gastric cancer infiltrated the serosa **(A)**. Gastroscopy showed ulcerative lesions in the gastric body **(B)**. Postoperative histopathology (HE staining 10×10 times) suggested poorly differentiated gastric adenocarcinoma (pathological stage: T4) **(C)**.

**Figure 3:**
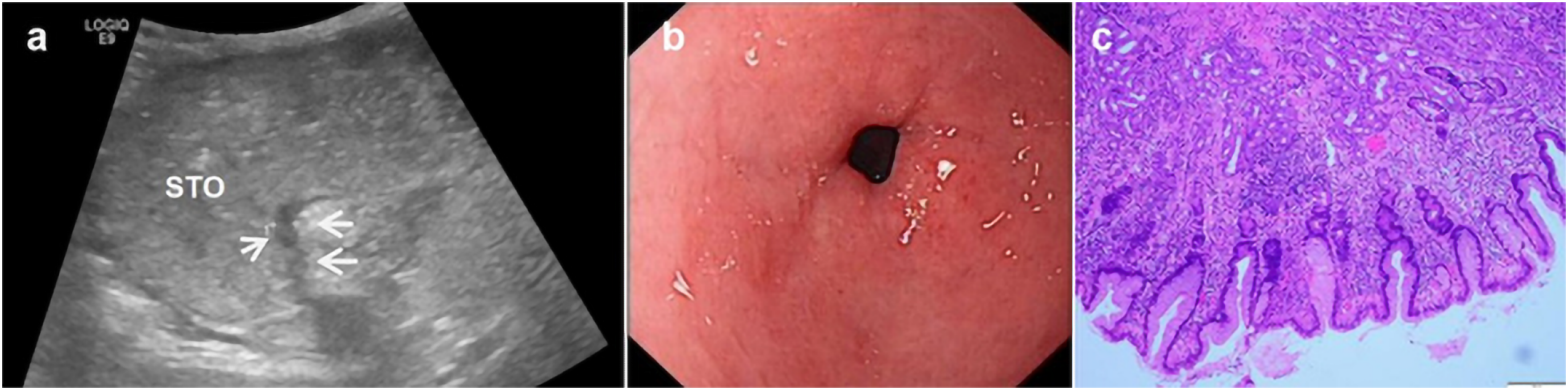
A 50-year-old woman underwent physical examination in our hospital. Oral contrast-enhanced ultrasonography showed a gastric wall thickness of 6.3 mm in the gastric antrum **(A)**. Gastroscopy showed congestion and flushing of the gastric antrum mucosa with scattered pox-like erosion **(B)**. The pathology of the gastroscopic biopsy (HE staining 10×10 times) suggested erosive gastritis with intestinal metaplasia **(C)**.

**Figure 4:**
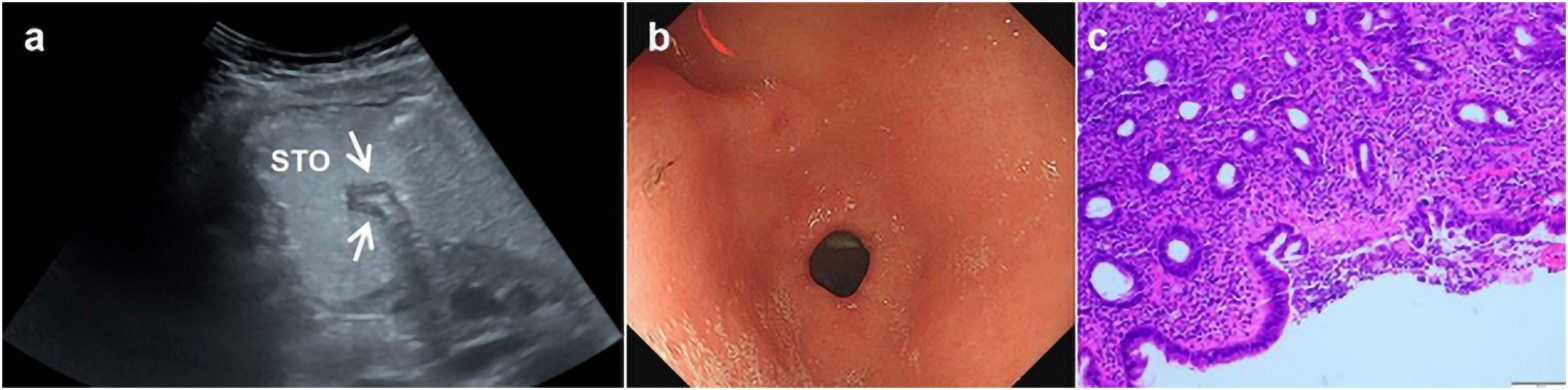
A 35-year-old woman underwent physical examination in our hospital. Oral contrast-enhanced ultrasonography showed a gastric wall thickness of 4.2 mm in the gastric antrum **(A)**. Gastroscopy showed congestion of the gastric antrum mucosa **(B)**. The pathology of the gastroscopic biopsy (HE staining 10×10 times) suggested superficial gastritis **(C)**. *Oral contrast-enhanced ultrasonography **(A)**; gastroscopy **(B)**; HE staining **(C)**.

Between January 2015 and March 2020, there were16988 individuals underwent physical examination with O-CEUS at the Physical Examination Center of our hospital, according to the inclusion and exclusion criteria, 12941 individuals were excluded due to insufficient follow-up duration, or incomplete follow-up data, or history of gastric cancer and gastric surgery. Finally, a total population of 4,047 were ultimately enrolled in this study (**Table 1**). There are 1676 males and 2371 females among them. Their median age was 55 years old (range: 19-93 years old). According to the thickness of the gastric wall, there were 3966 people in Group A, 47 in Group B, and 34 in Group C, respectively. During the follow-up period, GC occurred in 7 patients, and the incidence rate was 0.17%. Among them, one case occurred in Group A, two cases occurred in Group B, and four cases occurred in Group C (**Figures 2**–**4**).

**Table 1:** Characteristic data of different groups according to the thickness of the gastric wall.

Group	Number of Cases	Age median (range)	Gender: (Male/Female)	Gastric wall thickness (mm)	Incidence of gastric cancer	Months of Follow-up median (range)	Time of gastric cancer event(months)	Endoscopy (proportion)
**Group A** **(<6mm)**	3966	55 (19-93)	1634/2332	4.25±0.54	1	38 (36-95)	36	1322 (33.33%)
**Group B** **(6-9mm)**	47	52 (26-85)	20/27	6.83±0.73	2	41 (10-73)	18 (10-36)	47 (100%)
**Group C (>9mm)**	34	61 (39-84)	22/12	13.90±5.50	4	37 (24-67)	36 (24-48)	34 (100%)

### 3.2 Kaplan–Meier survival analysis

**Figure 5:**
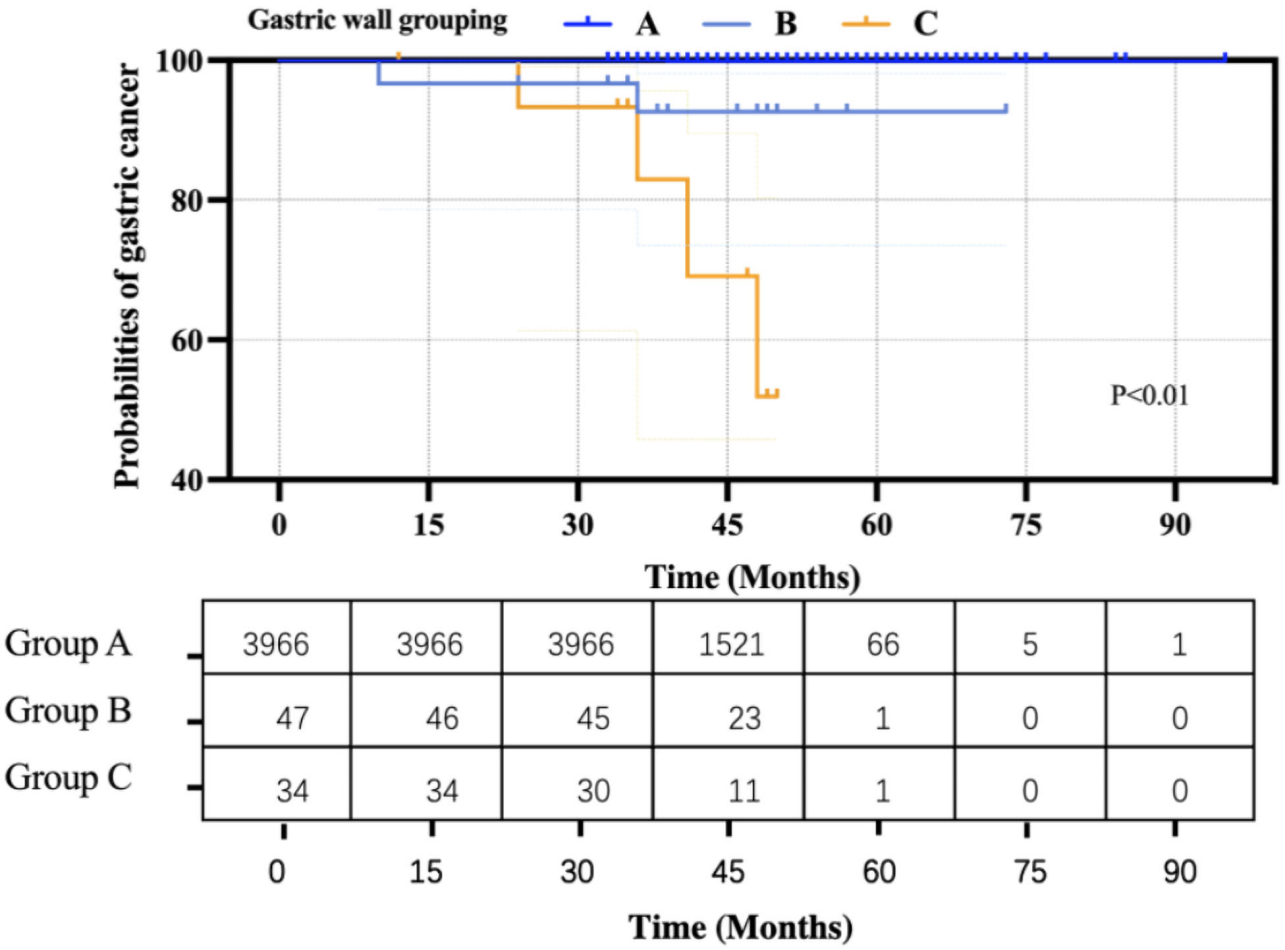
Kaplan–Meier estimates of the cumulative probabilities of gastric cancer events.

Based on the Kaplan–Meier curve and hazard function at the mean of covariates, the curves of the three groups were significantly different (P < 0.01) (**Figure 5**). Moreover, Cox’s regression analysis showed that people in Group B had a 1.50E+2-fold higher risk probability than that in Group A, and people in Group C had a 4.76E+2-fold higher risk probability than that in Group A.

## 4 Discussion

Gastroscopy, a preferred method for GC screening, has been widely accepted worldwide. However, considering its invasiveness and high cost, gastroscopy is not suitable for GC screening in a large population. Even in Japan, where a national GC screening program has been launched for a long time, gastroscopy is not recommended as a large-scale GC screening tool in the general population. There are some other noninvasive GC screening methods, such as biological markers, X-ray gastric imaging, or contrast-enhanced computed tomography. However, they are not considered to be the ideal routine screening methods due to their low sensitivity, high cost, or radiation exposure (18, 19). Therefore, they have limited value in GC screening (20, 21). For China, as a developing country with a high incidence of GC and a large population, it is necessary to carry out GC screening as early as possible. However, due to the limitations of people’s health concepts, imbalanced medical resources, and cost-effectiveness, it is difficult to conduct large-scale screening for gastroscopy. Therefore, it is necessary to develop a convenient, effective, inexpensive, and noninvasive method to optimize the GC screening system. O-CEUS is one kinds of methods that might be more acceptable for the GC screening of a large population. There are several advantages to O-CEUS use in GC screening. Firstly, the O-CEUS procedure is simple. The people only needed to drink the O-UCAs solution before examination without any other special preparations. Secondly, the O-CEUS procedure is convenient as it could be carried out by conventional ultrasound machines, which helps to carry out in community hospitals and physical examination center. Thirdly, the requirement for the operator’s experience of O-CEUS is low. According to our experience, operators can perform the O-CEUS procedure independently after one week’s standard training. What’s more, the cost of O-CEUS is relatively lower (approximately 300 RMB) when compared with gastroscopy (approximately 1000 RMB). Nowadays, the newly Expert Consensus on China’s Early Gastric Cancer Screening Process (2017, Shanghai) stated that O-CEUS could be used as a routine imaging examination for the screening of GC (22, 23).

Previous studies have shown that the gastric wall thickness assessed by O-CEUS is helpful for the diagnosis and evaluation of gastric diseases (24–29). Liu et al. (15) reported the establishment of a stomach ultrasound report and data system (Su-RADS) using O-CEUS for GC screening. Their findings suggested that the occurrence of GC could be predicted by gastric wall thickness. However, their Su-RADS was not practical enough because the thickness of multiple sites needed to be evaluated and the differences between the thickness of adjacent categories were only 1-2 mm. Moreover, subjective measurement errors occurred often. In this study, we retrospectively analyzed the use of O-CEUS for GC screening. According to our previous research and literature reports (14, 17, 22), we established our stratification prediction system for the occurrence of GC based on the thickness of the gastric wall. The people were divided into three groups with cutoff values of 6 mm and 9 mm. Based on the Kaplan–Meier curve and hazard function at the mean of covariates, the curves of the three groups were significantly different (P < 0.01), indicating that the risk of GC occurrence was different among them, with Group C having the highest risk of GC occurrence. In our opinion, this stratification prediction system is simple, easy to understand and use. It is suitable to use as a screening tool for GC at physical examination centers. At the same time, different follow-up systems and screening strategies can be established according to this grouping. For the people in Group A, if there are no high-risk factors such as a history of digestive diseases or a family history of GC, a regular physical examination every year can be considered without further gastroscopy. For the people in Group B, their risk of GC occurrence was 1.50E+2-fold higher than that in Group A. Gastroscopy is recommended to evaluate gastric diseases, including gastritis. According to the examination results, people should generally be followed up every half a year or every year. For people in Group C, their risk of GC occurrence was 4.76E+2-fold higher than that in Group A. Due to a high risk of GC occurrence, gastroscopy and biopsy should be performed immediately, and it is recommended to seek medical advice from a specialist. This strategy is useful for preliminary screening for GC, reducing unnecessary gastroscopies, and enabling populations at risk of GC to undergo gastroscopy in time to avoid missing a diagnosis and treatment.

There were some limitations in this study. First, this was a retrospective single-center study, and selection bias was unavoidable. A prospective multicenter study with a larger population is necessary in the future. Second, there were few GC cases in this study, the populations undergoing physical examinations reflected a “real-world” situation. Third, not all enrolled individuals underwent O-CEUS confirmed by gastroscopy or pathological results, since this study enrolled the population undergoing physical examination. Nevertheless, all the included individuals were required to be followed up for at least three years, and gastroscopy was performed for one-third of the populations in this study, including all people in Group B and C, which makes it possible to minimize the missed diagnosis.

In conclusion, O-CEUS is a convenient, economical, safe, and noninvasive screening method for GC. Measuring the thickness of the gastric wall is helpful in predicting the risk of GC occurrence by a stratification screening system, which is suitable for broad application in large populations.

## Data availability statement

The original contributions presented in the study are included in the article/supplementary material. Further inquiries can be directed to the corresponding authors.

## Ethics statement

The studies involving humans were approved by The Eighth Affiliated Hospital of Sun Yat-sen University. The studies were conducted in accordance with the local legislation and institutional requirements. The ethics committee/institutional review board waived the requirement of written informed consent for participation from the participants or the participants’ legal guardians/next of kin because This research has been approved by the Institutional Review Board of hospital. As a retrospective study, the informed consents of all participants were waived in this study.

## Author contributions

XJ, YF, and FJ contributed to the study designed. GN, CM, CQ, XN, TW and YF contributed to data collection. GN, YH, ZX, ZH, and HY contributed to data analysis. GN and XJ contributed to statistical analyses. GN, YH and XJ contributed to manuscript writing. All authors contributed to the article and approved the submitted version.
